# ULTRAPROCESSED FOODS AND COGNITIVE FUNCTIONING IN OLDER ADULTS

**DOI:** 10.1093/geroni/igad104.2139

**Published:** 2023-12-21

**Authors:** Elayna Seago, Benjamin Katz

**Affiliations:** Virginia Tech, Blacksburg, Virginia, United States; Virginia Tech, Blacksburg, Virginia, United States

## Abstract

Consumption of ultra-processed foods (UPFs) is increasing globally. Previous research has explored the psychological effects of this dietary shift, including associations between diet and cognition. While this previous work shows that neuroprotective dietary patterns in general, and UPF consumption in particular, may have a deleterious effect on cognition, less research has explored the specific cognitive domains most closely associated with UPF consumption. The present study draws from the 2016 Harmonized Cognitive Assessment Protocol and the 2012 Health Care and Nutrition Study (HCNS) from the Health and Retirement Study to examine the relationship between UPF consumption and a wide a wide array of fluid and memory-based cognitive measures in older adults. Additionally, we used the food frequency questionnaire from the HCNS to categorize participants’ foods as ultra-processed using the NOVA system. Initial analyses using analysis of covariance with UPF bins, controlling for age, gender, and self-rated health, indicates that measures of processing speed, but not other fluid measures nor most measures of delayed recall, were most closely associated with UPF consumption. These initial findings provide more specific detail regarding the cognitive correlates of UPF-heavy diets and provide a pathway to future work that more closely examines the longitudinal effects and underlying physiological mechanisms of UPF consumption.

